# Postlaparoscopic appendectomy acute pain: identifying risk factors and building a clinical prediction model

**DOI:** 10.3389/fsurg.2026.1793752

**Published:** 2026-04-22

**Authors:** Yubo Zhang, Dake Liu, Dongdong Wang, Ying Chen, Lin Li, Jionghui Fu, Weibo Liu, Jingcheng Zhang

**Affiliations:** Shijiazhuang People’s Hospital, Shijiazhuang, China

**Keywords:** acute pain, appendectomy, laparoscopy, postoperative, single-port, three-port

## Abstract

**Background:**

Laparoscopic appendectomy is the standard treatment for acute appendicitis; however, postoperative acute pain remains a significant challenge. This study aimed to identify risk factors and develop an externally validated nomogram to predict moderate-to-severe acute pain following the procedure.

**Methods:**

A retrospective study was conducted, including a training cohort (*n* = 430) and an independent external validation cohort (*n* = 124). Postoperative pain intensity was quantified using the peak numeric rating scale (NRS) score recorded within the first 24 h (assessed at 1, 3, 7, 9, 12, and 24 h). Patients were categorized into mild (NRS ≤ 3) and moderate-to-severe (NRS > 3) pain groups. Potential risk factors were identified via univariate analysis, and multivariable binary logistic regression was performed to determine independent predictors after assessing multicollinearity using the variance inflation factor. A nomogram-based predictive model was then developed and rigorously evaluated using the area under the curve (AUC), calibration plots, and decision curve analysis (DCA) in both cohorts.

**Results:**

Multivariable binary logistic regression identified three independent predictors of moderate-to-severe acute postoperative pain: surgical approach [three-port laparoscopic appendectomy (TPLA) vs. single-port laparoscopic appendectomy (SPLA); *P* < 0.01, odds ratio (OR) = 5.504; 95% CI 3.423–8.852], preoperative total delay (*P* = 0.005, OR = 1.496; 95% CI 1.129–1.983), and admission body temperature (*P* = 0.008, OR = 1.797; 95% CI 1.168–2.763). The developed nomogram exhibited robust discriminative performance, with an AUC of 0.762 (95% CI 0.716–0.808) in the training set and 0.785 in the external validation set. Calibration curves for both cohorts demonstrated optimal agreement between predicted and observed outcomes. In the validation cohort, DCA confirmed significant clinical net benefits across threshold ranges of 10%–14% and 16%–95%.

**Conclusion:**

Surgical approach, preoperative total delay, and admission body temperature were identified as independent predictors of acute pain following laparoscopic appendectomy. Compared with TPLA, the SPLA approach was associated with a significantly lower risk of moderate-to-severe acute pain. The externally validated nomogram provides a reliable clinical tool with high discriminative power and practical applicability, facilitating the identification of high-risk patients and supporting the optimization of individualized perioperative pain management strategies.

## Highlights

- Surgical approach, preoperative delay, and body temperature independently predict pain were identified as three independent predictors.
- Single-port laparoscopic appendectomy significantly reduces acute pain risk.
- The nomogram achieved robust performance with an area under the curve of 0.785 in external validation.
- Decision curve analysis (DCA) confirmed the model's clinical net benefit across wide threshold ranges.
- Preoperative delay is a critical modifiable factor for optimizing pain management.

## 1 Introduction

Appendicitis remains one of the most prevalent acute abdominal conditions, requiring immediate intervention across all age groups (1). The surgical management of appendicitis has undergone continuous refinement since the first appendectomy in the 18th century, progressing from open procedures to laparoscopic interventions, which have advanced from conventional three-port to single-incision methodologies (2, 3). In contemporary clinical practice, three-port laparoscopic appendectomy (TPLA) and single-port laparoscopic appendectomy (SPLA) have emerged as the predominant surgical approaches. While minimally invasive techniques have effectively alleviated surgical trauma, postoperative acute pain remains an inevitable clinical challenge.

As a predominant acute nociceptive emergency, postoperative pain typically peaks within 72 h, has a median duration of 7 days, and resolves within 30 days in most patients (4). Despite an enhanced understanding of pain pathophysiology and the continuous development of preventive strategies, the incidence of moderate-to-severe postoperative acute pain remains high (5). Inadequate management not only extends the length of hospitalization and elevates healthcare expenditures but also impedes rehabilitation and heightens patient anxiety, potentially leading to the transition from acute to chronic pain (6, 7). The 2023 China Acute Postoperative Pain Study reported a 48.7% incidence rate of moderate-to-severe pain on the first postoperative day (8).

Consequently, systematically identifying the risk factors associated with acute pain after laparoscopic appendectomy is pivotal for mitigating postoperative suffering, enhancing therapeutic adherence, and accelerating recovery (9). While previous research has explored the impact of preoperative delays on the risk of complicated appendicitis, the specific determinants of postoperative acute pain intensity—and their integration into a validated predictive framework—require further investigation. A clinical nomogram, as a visual and intuitive tool, offers a robust method for individualizing risk assessment and guiding perioperative management.

Consequently, this study was designed to investigate the predictive determinants of moderate-to-severe acute pain in patients undergoing laparoscopic appendectomy. The objective was to construct and, importantly, externally validate a nomogram-based prediction model to provide evidence-based guidance for pain prophylaxis and individualized clinical decision-making.

## 2 Methods

### 2.1 Study setting

A retrospective cohort study was conducted to identify risk factors and develop a clinical prediction model for acute pain following laparoscopic appendectomy. The research protocol was formally approved by the Medical Research Ethics Committee of Shijiazhuang People's Hospital (Approval No. 2025-092). Due to the retrospective design of the study, the requirement for informed consent was waived. All methods were conducted in accordance with the relevant guidelines and regulations. This work is reported in accordance with the STROCSS criteria (10).

The study population was divided into two distinct cohorts based on hospital campuses to ensure geographic and systemic validation:

1. Training cohort: A total of 430 patients who underwent laparoscopic appendectomy at the Jianhua District of Shijiazhuang People's Hospital from May 2023 to March 2025 were included to construct the predictive nomogram.
2. External validation cohort: To evaluate the generalizability of the model's, an independent cohort of 124 patients treated at the Fanxi Road District of Shijiazhuang People's Hospital during the same time period was included.

### 2.2 Inclusion and exclusion criteria

The inclusion criteria are as follows:.

(1) Patients meeting the clinical diagnostic criteria for appendicitis and undergoing laparoscopic intervention (11); (2) patients or legal guardians who voluntarily selected the surgical approach (TPLA or SPLA); (3) patients who did not undergo any other abdominal surgeries during the same hospitalization; and (4) availability of complete clinical, laboratory, and operative data.

The exclusion criteria are as follows:

(1) Patients with severe coagulation disorders or poor cardiopulmonary function who are unable to tolerate surgery or anesthesia; (2) patients unable to comply with clinical treatment; (3) patients unable to adhere to follow-up after surgery; and (4) female reproductive system conditions that could confound assessment, including pregnancy.

A total of 119 cases were excluded during the initial screening across both sites, resulting in a finalized dataset for rigorous analysis.

### 2.3 Outcome definition and grouping

The primary outcome of this research was the intensity of acute postoperative pain within the initial 24 h following the surgical procedure. Pain levels were quantified using the numeric rating scale (NRS), which has demonstrated superior clinical utility and patient compliance compared to other visual scales in the context of acute surgical care.

To comprehensively capture of the pain experience of the patient and to account for fluctuations in analgesic efficacy, a dynamic assessment protocol was implemented. NRS scores were systematically recorded by trained nursing staff at 1, 3, 7, 9, 12, and 24 h postoperatively. For statistical modeling and risk stratification, the peak NRS score—defined as the highest value recorded across all six assessment time points—was used as the definitive measure of pain intensity for each patient.

Patients were subsequently categorized into two distinct groups based on the clinical severity of their peak pain:

1. Mild pain group: Patients with a peak NRS ≤ 3, indicating manageable pain that typically does not significantly interfere with early mobilization or recovery.
2. Moderate-to-severe pain group: Patients with a peak NRS > 3, representing pain levels that generally require enhanced analgesic intervention and may impede the recovery process.

This dichotomous classification served as the dependent variable in subsequent univariate and multivariable logistic regression analyses.

### 2.4 Surgical equipment

#### 2.4.1 Laparoscopic system

The surgical procedures were conducted using two high-definition laparoscopic systems: the STORZ laparoscopic system (Karl STORZ, Tuttlingen, Germany) and the DPM laparoscopic system (Beijing Precision Digital Medical Technology Co., Ltd., Beijing, China). Both systems provided adequate visualization and resolution for the precise execution of the surgical approach.

#### 2.4.2 Disposable multi-channel single-port laparoscopic surgical puncture device

For patients undergoing SPLA, a specialized disposable multi-channel single-port laparoscopic surgical puncture device was used. This device, supplied by Mindray Medical (Shenzhen, China; batch number: 104279), was designed to facilitate the simultaneous insertion of multiple laparoscopic instruments through a single transumbilical access site.

### 2.5 Surgical procedure

#### 2.5.1 TPLA surgical procedure

(1) Under anesthesia, the patient was positioned in a supine manner. A 10-mm incision was made above the umbilicus, and a 10-mm disposable trocar was inserted to establish a carbon dioxide pneumoperitoneum at a pressure of 12 mmHg. A laparoscope was then inserted to inspect for any potential organ injury beneath the puncture site. (2) A 5-mm incision was made at the midpoint between the umbilicus and the pubic symphysis, and an additional a 10-mm incision was made on the lateral edge of the right rectus abdominis muscle. Under laparoscopic surveillance, disposable trocars and instruments were inserted through these sites to locate the appendix and examine the extent of inflammation and surrounding adhesions. (3) After identifying the base of the appendix, the mesentery of the appendix was dissected at a vascular-free area on the mesenteric side and then clamped with Hemlock clips and cut. At a distance of 0.5 cm from the base of the appendix, a double ligation was performed using a snare and Hemlock clips before cutting; the specimen was removed through the 10-mm trocar. (4) Based on the extent of inflammatory purulence observed, saline irrigation of the abdominal cavity was considered. Hemostasis was confirmed, and the laparoscopic instruments were withdrawn. Carbon dioxide was then evacuated from the abdominal cavity. (5) The abdominal incisions were closed in layers using absorbable sutures to ensure proper wound healing.

#### 2.5.2 SPLA surgical procedure

(1) After induction of anesthesia, the patient was placed in a supine position. An approximately 20-mm-long incision was made above the umbilicus, and the abdominal cavity was sequentially accessed. A single-port laparoscopic setup was inserted, and carbon dioxide was used to establish a pneumoperitoneum at a pressure of 12 mmHg. All procedures were performed through this incision. (2) The subsequent steps were identical to those performed in the TPLA group.

### 2.6 Postoperative management

Postoperative recovery after LA is typically rapid due to its minimally invasive nature. To optimize clinical outcomes and ensure patient safety, a standardized perioperative care pathway was followed for all patients. An evidence-based enhanced recovery after surgery protocol was routinely utilized, integrating multimodal strategies for clinical management (12, 13). Vital signs were continuously monitored until hemodynamic and clinical stability were achieved. Metabolic homeostasis was maintained by administering intravenous fluids and nutritional support as clinically indicated. Early mobilization was encouraged, as tolerated, to promote functional recovery and minimize the risk of thromboembolic complications.

On the first postoperative day, routine laboratory evaluations—including complete blood count and metabolic panels—were performed to monitor early recovery and detect potential complications. Following hospital discharge, a structured follow-up was conducted by designated personnel at 1 week postoperatively, with subsequent outpatient assessments scheduled as clinically indicated.

### 2.7 Observational indicators

#### 2.7.1 Preoperative indicators

Preoperative data included demographic characteristics, clinical history, and laboratory assessments:

1. Demographics and history: Gender, age, body mass index (BMI), and history of previous abdominal surgery, hypertension, diabetes, smoking, and drinking.
2. Clinical presentation: Admission body temperature and preoperative total delay (14–16), defined as the cumulative duration from the onset of symptoms to the initiation of the surgical procedure.
3. Laboratory metrics: Complete blood count parameters, including white blood cell (WBC) count, neutrophil (NEUT) count, lymphocyte (LYMPH) count, and platelet (PLT) count. Calculated systemic markers included the neutrophil-to-lymphocyte ratio (NLR) and the systemic immune-inflammation index (SII). The SII was calculated as PLT × NEUT/LYMPH and used as a comprehensive indicator of systemic immune and inflammatory status. Serum albumin (ALB) levels were also recorded as part of the routine preoperative metabolic panel to assess baseline physiological reserve.

#### 2.7.2 Intraoperative indicators

Intraoperative parameters focused on the technical aspects and findings of the surgical intervention:

1. Surgical approach: The primary variable of interest was the type of procedure performed, specifically TPLA and SPLA.
2. Operative details: Estimated blood loss and operative time.
3. Pathological findings: Dimensions of the resected appendix, including appendiceal length and width.

### 2.8 Statistical analysis

Statistical analyses were performed using SPSS version 27.0 and R version 4.2.2. A two-sided *P* < 0.05 was considered statistically significant. Continuous variables were expressed as mean ± standard deviation ($\bar{x}\pm s$) or median with interquartile range and were compared using Student's *t*-test or the Mann–Whitney *U*-test. Categorical variables were presented as frequencies and percentages and were analyzed using the chi-square test or Fisher's exact test.

Prior to multivariable analysis, the variance inflation factor (VIF) was calculated to assess multicollinearity among variables. Variables with a VIF > 5 were excluded to ensure model stability. Variables with *P* < 0.05 in the univariate analysis were then incorporated into a multivariable binary logistic regression model using the backward stepwise method to identify independent predictors.

Based on the independent predictors identified in the development cohort (Jianhua District), a nomogram was constructed. The predictive performance of the model was then evaluated in both the development cohort and the independent external validation cohort (Fanxi Road District). Discrimination was assessed using the area under the curve (AUC), calibration was evaluated via calibration plots, and clinical utility was measured using decision curve analysis (DCA).

## 3 Results

### 3.1 Baseline characteristics and univariate analysis

A total of 554 patients who met the inclusion and exclusion criteria were enrolled in this study. The study population was geographically stratified into a development cohort comprising 430 patients from the Jianhua District and an independent external validation cohort comprising 124 patients from the Fanxi Road District.

Within the development cohort, the overall incidence of moderate-to-severe acute postoperative pain (peak NRS > 3) was 52.56% (226/430). The baseline demographic, preoperative, and intraoperative characteristics of the development cohort, stratified by pain severity (mild pain compared with moderate-to-severe pain), are detailed in Tables 1, 2.

**Table 1:** Baseline demographic and preoperative characteristics of patients in the development cohort stratified by pain severity.

Variable	Mild pain group (*n* = 204)	Moderate-to-severe pain group (*n* = 226)	Statistical test values	*P-*value
Gender [*n* (%)]			3.165	0.075
Men	89 (43.63%)	118 (52.21%)		
Women	115 (56.37%)	108 (47.79%)		
Age ($\bar{x}\pm s$, years)	26.87 ± 14.32	28.29 ± 14.29	1.275	0.202
BMI ($\bar{x}\pm s$, kg/m^2^)	22.51 ± 4.13	23.81 ± 4.94	2.575	0.010
Previous history of abdominal surgery [*n* (%)]			2.284	0.131
Yes	20 (9.80%)	33 (14.60%)		
No	184 (90.20%)	193 (85.40%)		
Hypertension [*n* (%)]			0.466	0.495
Yes	8 (3.92%)	12 (5.31%)		
No	196 (96.08%)	214 (94.69%)		
Diabetes [*n* (%)]			4.352	0.037
Yes	8 (3.92%)	2 (0.88%)		
No	196 (96.08%)	224 (99.12%)		
Smoking history [*n* (%)]			0.295	0.587
Yes	21 (10.29%)	27 (11.95%)		
No	183 (89.71%)	199 (88.05%)		
Drinking history [*n* (%)]			1.116	0.291
Yes	18 (8.82%)	27 (11.95%)		
No	186 (91.18%)	199 (88.05%)		
Preoperative total delay [*n* (%)]			6.755	0.034
0–24h	69 (33.82%)	51 (22.57%)		
24–72h	52 (25.49%)	67 (29.65%)		
＞72h	83 (40.69%)	108 (47.78%)		
WBC count ($\bar{x}\pm s$, 10^9^/L)	8.83 ± 4.06	10.49 ± 4.82	3.793	<0.01
NEUT count ($\bar{x}\pm s$, 10^9^/L)	6.41 ± 4.19	8.23 ± 4.81	4.214	<0.01
LYMPH count ($\bar{x}\pm s$, 10^9^/L)	1.70 ± 0.78	1.51 ± 0.79	2.548	0.011
PLT count ($\bar{x}\pm s$, 10^9^/L)	256.36 ± 67.66	245.90 ± 70.56	1.446	0.148
NLR ($\bar{x}\pm s$)	5.78 ± 7.20	9.65 ± 13.76	4.268	<0.01
SII ($\bar{x}\pm s$, 10^9^/L)	1,452.25 ± 1,814.53	2,269.74 ± 3,253.53	3.837	<0.01
ALB level ($\bar{x}\pm s$, g/L)	44.03 ± 3.30	43.33 ± 3.64	1.593	0.111
Admission body temperature ($\bar{x}\pm s$, ℃)	36.62 ± 0.46	36.85 ± 0.69	3.916	<0.01

**Table 2:** Intraoperative and pathological characteristics of patients in the development cohort stratified by pain severity.

Variable	Mild pain group (*n* = 204)	Moderate-to-severe pain group (*n* = 226)	Statistical test values	*P-*value
Surgical approach [*n* (%)]			71.544	<0.01
SPLA	141 (69.12%)	64 (28.32%)		
TPLA	63 (30.88%)	162 (71.68%)		
Blood loss ($\bar{x}\pm s$, mL)	5.44 ± 3.49	6.52 ± 5.84	1.779	0.075
Appendiceal length ($\bar{x}\pm s$, cm)	7.29 ± 1.45	7.56 ± 1.72	1.607	0.108
Appendiceal width ($\bar{x}\pm s$, cm)	0.84 ± 0.21	0.93 ± 0.26	4.148	<0.01
Operative time ($\bar{x}\pm s$, min)	66.13 ± 20.79	71.85 ± 25.95	2.088	0.037

Univariate analysis revealed that patients in the moderate-to-severe pain group exhibited significantly different clinical profiles compared with those in the mild pain group. Among preoperative characteristics, significant differences were observed in BMI (*P* = 0.010) and the prevalence of diabetes (*P* = 0.037). Significant variations were also noted in admission body temperature (*P* < 0.01) and preoperative total delay (*P* < 0.05). Laboratory markers indicative of systemic inflammation, including WBC count, NEUT count, LYMPH count, NLR, and SII, were all statistically significant different between the two groups (all *P*'s < 0.05). Regarding intraoperative parameters, the choice of surgical approach was highly significant: a substantially greater proportion of patients undergoing TPLA experienced moderate-to-severe pain compared with those undergoing SPLA (*P* < 0.01). In addition, appendiceal width (*P* < 0.01) and operative time (*P* = 0.037) were also found to be significantly associated with the severity of acute postoperative pain.

### 3.2 Predictor selection and multivariable analysis

To identify independent risk factors for moderate-to-severe acute postoperative pain, variables demonstrating statistical significance (*P* < 0.05) in the univariate analysis were incorporated into the multivariable analysis. Prior to the construction of the predictive model, collinearity diagnostics were conducted to assess potential multicollinearity among candidate variables, and the results are presented in Table 3. Significant multicollinearity was identified among several systemic inflammatory markers, specifically WBC count (VIF = 62.764), NEUT count (VIF = 72.266), NLR (VIF = 20.910), and SII (VIF = 22.896). To ensure the mathematical stability and interpretability of the model, these variables were excluded from further multivariable analysis.

**Table 3:** Collinearity diagnostics of candidate variables for moderate-to-severe acute postoperative pain.

Variable	Unstandardized coefficients		Standardized coefficients	*t*	*P*	Collinearity statistics	
	β	Std. error	Beta			Tolerance	VIF
(Constant)	−10.211	4.155		−2.458	0.014		
Gender	−0.242	0.133	−0.088	−1.820	0.070	0.713	1.402
Age	0.002	0.006	0.024	0.418	0.676	0.498	2.007
BMI	0.011	0.015	0.038	0.780	0.436	0.701	1.426
Previous history of abdominal surgery	0.078	0.189	0.019	0.412	0.680	0.813	1.230
Hypertension	0.171	0.324	0.026	0.529	0.597	0.677	1.477
Diabetes	0.107	0.442	0.012	0.243	0.808	0.709	1.410
Smoking history	0.008	0.246	0.002	0.034	0.973	0.523	1.910
Drinking history	−0.204	0.254	−0.045	−0.802	0.423	0.530	1.886
Preoperative total delay	0.210	0.073	0.127	2.873	0.004	0.842	1.188
WBC count	0.070	0.098	0.231	0.718	0.473	0.016	62.764
NEUT count	−0.044	0.103	−0.146	−0.423	0.672	0.014	72.266
LYMPH count	0.137	0.147	0.079	0.937	0.349	0.235	4.253
PLT count	−0.001	0.001	−0.044	−0.720	0.472	0.443	2.259
NLR	0.036	0.023	0.292	1.570	0.117	0.048	20.910
SII	0.000	0.000	−0.202	−1.036	0.301	0.044	22.896
ALB level	0.007	0.018	0.018	0.379	0.705	0.775	1.291
Admission body temperature	0.302	0.108	0.132	2.796	0.005	0.741	1.350

Dependent variable: NRS score.

The remaining non-collinear variables were then analyzed using a multivariable binary logistic regression model. As detailed in Table 4, three variables were identified as robust independent predictors of moderate-to-severe acute pain:

(1) Surgical approach: Patients undergoing SPLA had a significantly higher risk than those undergoing TPLA (OR = 5.504, 95% CI: 3.423–8.852, *P* < 0.01).
(2) Preoperative total delay: A prolonged duration from symptom onset to surgery independently increased the risk of severe pain (OR = 1.496, 95% CI: 1.129–1.983, *P* = 0.005).
(3) Admission body temperature: Elevated baseline body temperature also served as an independent risk factor for higher postoperative pain intensity (OR = 1.797, 95% CI: 1.168–2.763, *P* = 0.008).

**Table 4:** Multivariable logistic regression analysis of independent predictors for moderate-to-severe acute postoperative pain.

Variable	*β*	Standard error	*P*	OR	95%CI	
					Lower limit	Upper limit
Surgical approach	1.706	0.242	<0.01	5.504	3.423	8.852
Preoperative total delay	0.403	0.144	0.005	1.496	1.129	1.983
Admission body temperature	0.586	0.220	0.008	1.797	1.168	2.763

### 3.3 Model development and validation

**Figure 1:**
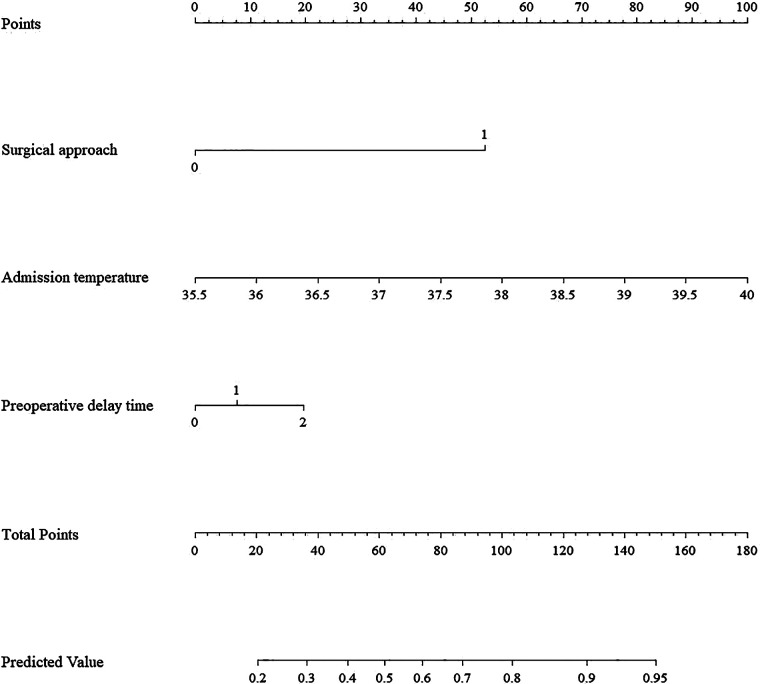
Nomogram for predicting moderate-to-severe acute postoperative pain after laparoscopic appendectomy.

Based on the three independent predictors identified in the multivariable analysis, a clinical nomogram was constructed to facilitate the individualized risk estimation of moderate-to-severe acute pain following laparoscopic appendectomy. The model and its performance metrics are presented in Figure 1.

**Figure 2:**
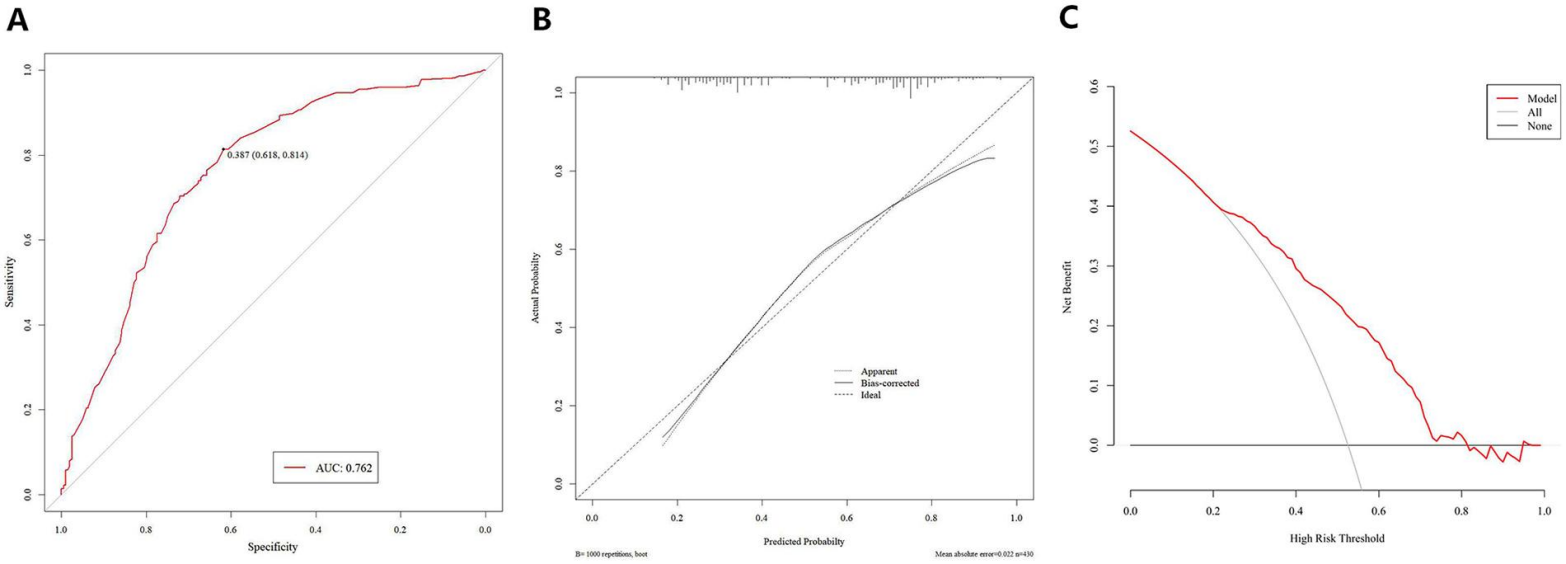
Performance evaluation of the nomogram in the development cohort. **(A)** ROC curve for assessing the discriminative ability of the model; **(B)** calibration plot demonstrating the agreement between predicted probabilities and actual observed outcomes of moderate-to-severe pain; and **(C)** DCA illustrating the net benefit and clinical utility of the nomogram across a range of threshold probabilities.

The predictive performance of the developed nomogram was comprehensively evaluated in the development cohort across three dimensions, as presented in Figure 2: (1) Discrimination: the model demonstrated robust discriminative ability with an AUC of 0.762 (95% CI: 0.718–0.806), yielding a sensitivity of 0.814 and a specificity of 0.618 at the optimal threshold, as illustrated in the ROC curve. The ROC curve is used in predictive models to evaluate the discrimination ability of binary classification models. It takes the false positive rate (the proportion of negative cases wrongly classified as positive cases) as the x–axis and the true positive rate (the proportion of correctly identified positive cases) as the y–axis. By changing the judgment threshold, it depicts the overall performance trajectory of the model; (2) Calibration: the calibration plot exhibited good agreement between predicted probabilities and actual observed outcomes of moderate-to-severe pain, indicating high predictive accuracy of the nomogram; (3) Clinical utility: DCA quantified the clinical value of the model and showed a positive net benefit across threshold probabilities of 25%–80%, confirming the high applicability of the model in clinical decision-making contexts.

**Figure 3:**
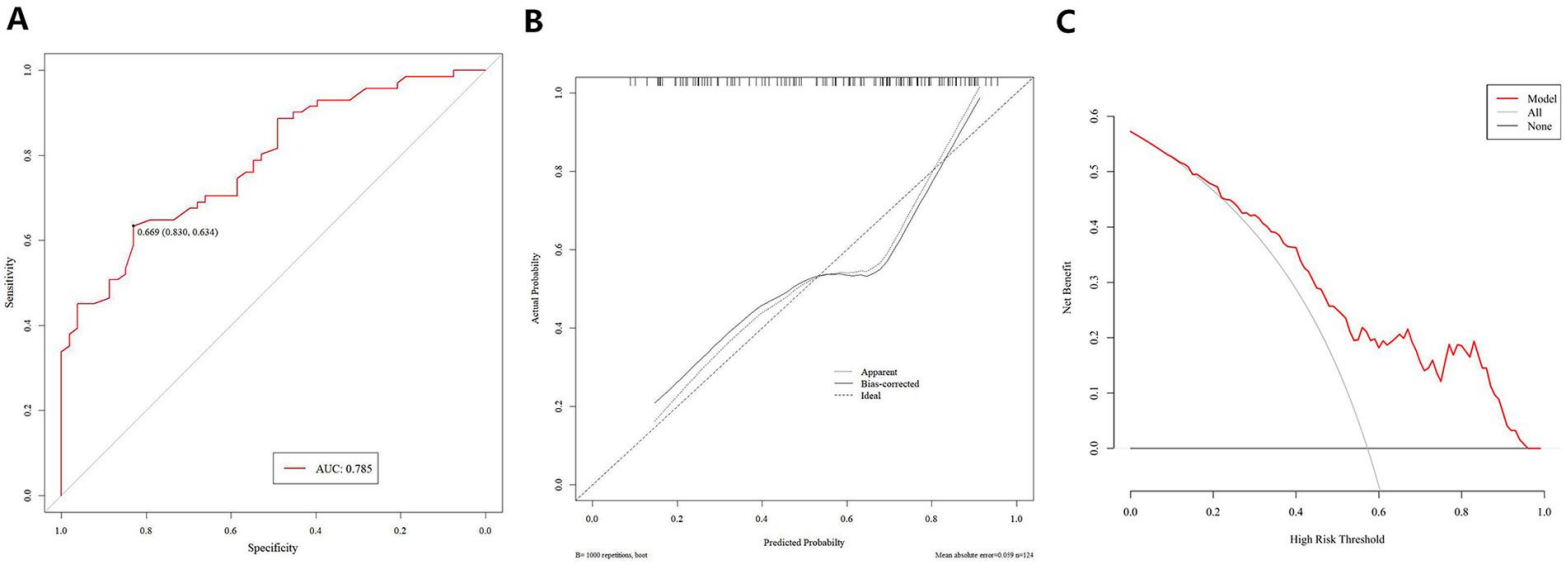
Performance evaluation of the nomogram in the external validation cohort. **(A)** ROC curve for assessing the discriminative ability of the model; **(B)** calibration plot demonstrating the agreement between predicted probabilities and actual observed outcomes of moderate-to-severe pain; and **(C)** DCA illustrating the net benefit and clinical utility of the nomogram across a range of threshold probabilities.

In the external validation cohort, the nomogram maintained exceptional predictive performance, as presented in Figure 3. (1) Discrimination: the model achieved an AUC of 0.785 (95% ci: 0.704–0.866), yielding a sensitivity of 0.634 and a specificity of 0.830 at the optimal threshold, as illustrated by the ROC curve; (2) Calibration: the calibration plot exhibited good agreement between predicted probabilities and actual observed outcomes of moderate-to-severe pain, indicating robust calibration and generalization of the nomogram; (3) Clinical utility: DCA quantified the clinical value of the model and showed a positive net benefit across threshold probabilities of 10%–14% and 16%–95%, confirming the high applicability of the model in external clinical settings.

## 4 Discussion

Although laparoscopic appendectomy has become the standard minimally invasive treatment for appendicitis, acute postoperative pain remains a prominent clinical challenge that can impede early rehabilitation and increase healthcare burden (17, 18). This study successfully developed and externally validated a clinical nomogram to individualize the risk assessment of moderate-to-severe acute pain following laparoscopic appendectomy. Multivariable analysis identified three robust independent predictors: surgical approach (OR = 5.504), prolonged preoperative total delay (OR = 1.496), and elevated admission body temperature (OR = 1.797). Addressing previous methodological limitations in prediction modeling, this study incorporated a rigorous external validation cohort. The nomogram demonstrated moderate-to-good discriminative ability, with an AUC of 0.762 in the development cohort and a robust AUC of 0.785 in the external validation cohort. Furthermore, the calibration plots and decision analysis curves confirmed its high applicability in clinical decision-making contexts, providing clinicians with a reliable and visually accessible tool to support perioperative pain management.

Among the identified predictors, the choice of surgical modality exhibited the most significant association with acute postoperative pain. Patients undergoing TPLA experienced a higher risk of moderate-to-severe pain compared with those undergoing SPLA. The analgesic superiority of SPLA can primarily be attributed to its more minimally invasive approach: conventional multiport laparoscopy requires additional trocars lateral to the ventral white line, which can inevitably increase collateral damage to abdominal wall nerves and muscles (19, 20). Furthermore, the minimized surgical access in single-port procedures may alleviate surgery-related psychological distress, thereby subconsciously modulating pain perception (21).

However, the markedly elevated odds ratio (OR = 5.504) associated with TPLA must be interpreted with caution. In retrospective cohorts, the allocation of surgical approach is inevitably influenced by baseline disease severity and surgeon preference. In real-world clinical practice, patients presenting with suspected complicated appendicitis, severe intra-abdominal adhesions, or extensive exudation are more likely to undergo TPLA to ensure optimal anatomical visualization and operational safety. Because the multivariable model in this study did not explicitly adjust for certain unmeasured intraoperative confounders, such as perforated or gangrenous status, the strong association observed between TPLA and elevated pain intensity might be partially confounded by the underlying severity of the appendicitis itself. Interestingly, while some previous literature has reported comparable pain outcomes between single-port and conventional multiport approaches, the present findings strongly align with recent studies suggesting a higher nociceptive burden associated with multiport laparoscopy in specific clinical scenarios (22). This implies that while the reduced access trauma of SPLA is advantageous, the choice of surgical modality must be rigorously individualized based on the specific pathological complexity of the patient.

In addition to the surgical approach, prolonged preoperative total delay and elevated admission body temperature were identified as significant independent risk factors. A longer delay typically reflects ongoing progression of the inflammatory process, leading to exacerbated local tissue edema and increased inflammatory exudation, which consequently sensitizes peripheral nerve endings (23). This observation is consistent with previous cohort studies emphasizing the pivotal role of prolonged preoperative inflammation in exacerbating postoperative pain sensitization (24–26). Similarly, an elevated admission body temperature serves as a direct marker of systemic inflammatory response (27). Severe inflammation not only aggravates tissue damage in the surgical area but may also lower the threshold of peripheral nociceptors through the release of cytokines, such as prostaglandins (28, 29). Notably, although multiple systemic inflammatory markers, including WBC and NEUT, demonstrated statistical significance in the univariate analysis, highly correlated variables were excluded based on VIF assessment prior to multivariable modeling to prevent the impact of multicollinearity from compromising the mathematical stability of the model, thereby ensuring the rigor of the final nomogram.

Several limitations of the present study should be acknowledged. First, given the retrospective design, although an independent external validation cohort was included to enhance methodological rigor and generalizability, the potential impact of unmeasured confounding factors on the predictive outcomes cannot be entirely ruled out. Second, the current pain assessment lacks a sufficiently detailed temporal dimension. To further improve the reproducibility of the study and comparability with other studies, future prospective studies need to more strictly standardize the timing of NRS assessments, for example by uniformly adopting the first postoperative day or a specific peak pain time. Finally, although the model demonstrated moderate-to-good predictive performance, it still requires validation with a more cautious approach in broader clinical settings before widespread implementation.

## 5 Conclusion

In conclusion, a clinical nomogram was successfully developed and externally validated for individualized risk prediction of moderate-to-severe acute pain following laparoscopic appendectomy. Three independent risk factors were identified: choice of surgical approach, elevated admission body temperature, and prolonged preoperative total delay. The model demonstrated moderate-to-good discriminative capacity and clinical utility, with an AUC of 0.762 in the development cohort and a robust AUC of 0.785 in the external validation cohort. By translating these perioperative parameters into an intuitive visual tool, this nomogram provides clinicians with a reliable and practical instrument to optimize risk stratification and implement personalized analgesic strategies in clinical practice.

## Data Availability

The raw data supporting the conclusions of this article will be made available by the authors, without undue reservation.
